# Content Analysis and Characterization of Medical Tweets During the Early Covid-19 Pandemic

**DOI:** 10.7759/cureus.13594

**Published:** 2021-02-27

**Authors:** Ross Prager, Michael T Pratte, Rudy R Unni, Sudarshan Bala, Nicholas Ng Fat Hing, Kay Wu, Trevor A McGrath, Adam Thomas, Brent Thoma, Kwadwo Kyeremanteng

**Affiliations:** 1 Department of Medicine, University of Ottawa, Ottawa, CAN; 2 Department of Medicine, McMaster University, Hamilton, CAN; 3 Department of Radiology, University of Ottawa, Ottawa, CAN; 4 Division of Critical Care, University of British Columbia, Vancouver, CAN; 5 Emergency Medicine, University of Saskatchewan, Saskatoon, CAN; 6 Division of Critical Care, University of Ottawa, Ottawa, CAN

**Keywords:** covid-19, social media, pandemic, free open access medical education, medical education, knowledge translation

## Abstract

Objective

The novel coronavirus disease 2019 (Covid-19) has infected millions worldwide and impacted the lives of many folds more. Many clinicians share new Covid-19-related resources, research, and ideas within the online Free Open Access to Medical Education (FOAM) community of practice. This study provides a detailed content and contributor analysis of Covid-19-related tweets among the FOAM community during the first months of the pandemic.

Design, Setting, and Participants

In this social media content analysis study, Twitter was searched from November 1, 2019, to March 21, 2020, for English tweets discussing Covid-19 in the FOAM community. Tweets were classified into one of 13 pre-specified content categories: original research, editorials, FOAM resource, public health, podcast or video, learned experience, refuting false information, policy discussion, emotional impact, blatantly false information, other Covid-19, and non-Covid-19. Further analysis of linked original research and FOAM resources was performed. One-thousand (1000) randomly selected contributor profiles and those deemed to have contributed false information were analyzed.

Results

The search yielded 8541 original tweets from 4104 contributors. The number of tweets in each content category were: 1557 other Covid-19 (18.2%), 1190 emotional impact (13.9%), 1122 FOAM resources (13.1%), 1111 policy discussion (13.0%), 928 advice (10.9%), 873 learned experience (10.2%), 424 non-Covid-19 (5.0%), 410 podcast or video (4.8%), 304 editorials (3.6%), 275 original research (3.2%), 245 public health (2.9%), 83 refuting false information (1.0%), and 19 blatantly false (0.2%).

Conclusions

Early in the Covid-19 pandemic, the FOAM community used Twitter to share Covid-19 learned experiences, online resources, crowd-sourced advice, and research and to discuss the emotional impact of Covid-19. Twitter also provided a forum for post-publication peer review of new research. Sharing blatantly false information within this community was infrequent. This study highlights several potential benefits from engaging with the FOAM community on Twitter.

## Introduction

Millions of cases of coronavirus disease 2019 (Covid-19) have been reported globally since the first known case in December 2019 [1-2]. Covid-19’s worldwide impact has been recognized through its classification as a global pandemic by the World Health Organization (WHO) [3]. Covid-19’s rapid spread has spurred healthcare workers, researchers, and members of the public to search for accurate and up-to-date information online. The rate of new Covid-19-related research, however, has challenged conventional methods of scientific knowledge dissemination (e.g. peer-reviewed journals), which do not always publish on rapid timelines [4]. In response, clinicians worldwide have turned to social media to debate new research while sharing their experiences and resources [5].

Social media use among clinicians is not a new phenomenon. In the past decade, an online community of practice has developed with the goal of sharing ideas, research, and learned experiences through freely published online resources [6-7]. Termed “Free Open Access to Medical Education” (FOAM), this movement has become a valuable resource for healthcare professionals and medical learners [7-8]. In addition to relaying explicit medical knowledge, it may also be an effective medium for transmitting tacit knowledge (experiential or process-based knowledge) [9]. Compared to traditional peer-reviewed journals, FOAM has variable publication and editorial processes relying heavily on post-publication peer review [10].

Optimizing knowledge translation is important during a pandemic, as critical decisions need to be made with limited evidence, and potentially practice-changing research can be published at any time. Within the FOAM community, Twitter is the most widely used social media platform to share ideas and discuss new research on Covid-19 [5]. On Twitter, contributors generate ‘tweets’ of up to 280 characters in length that can be tagged with searchable hashtags (#) and can include images, website links, and documents. While the important role of Twitter during Covid-19 has been recognized by the scientific community [5], a detailed characterization of its use, strengths, and limitations, including accuracy of content, is needed. This is particularly important for a publicly accessed platform like Twitter that may be susceptible to misleading or false information.

The objective of this study was to characterize Covid-19-related Twitter use by the FOAM community and to describe its content, trends, and contributors. In addition, the potential role of Twitter in spreading misinformation was assessed. This research represents an important first step in evaluating Twitter as a platform for knowledge translation during rapidly evolving healthcare crises.

## Materials and methods

Research ethics board approval for research involving publicly available data is not required at our institutions. Our protocol was registered on the Open Science Framework (OSF) prior to the initiation of data collection (https://osf.io/3tx96/). The original data are also published on OSF. The study has been reported in keeping with the Strengthening the Reporting of Observational Studies in Epidemiology (STROBE) statement [11]. Patients or the public were not involved in the design, conduct, reporting, or dissemination plans of our research.

Search strategy

We searched Twitter on March 21, 2020, for tweets with relevant hashtags from November 1, 2019 (the month preceding the first reported Covid-19 case in Wuhan, China) to March 21, 2020, using a commercially available hashtag collating tool, Tweet Binder (Pamplona, Spain). The period between November 2019 to March 2020 was chosen, as it represented a time period when little consolidated information on Covid-19 was available to healthcare professionals despite concern surrounding Covid-19 being high. Hashtags were selected by consensus of the authors, several of whom were clinician members of the FOAM community. Tweets were included if they contained both a hashtag commonly used by healthcare professionals to discuss FOAM topics (#FOAMed or #meded or #POCUS or #FOAMcc or #medtwitter) and a hashtag used to discuss Covid-19 (#Covid19 or #coronavirus or #Covid or #Covid-19). Alternatively, two Covid-19 FOAM-specific hashtags were also included (#Covid4MDs or #CovidFOAM). The search strategy was not case-sensitive.

Tweet analysis

We extracted the following data: total number of original tweets (original text or image content), retweets (a reposted tweet without modification), reach (number of unique people who saw the tweet), impressions (number of times a tweet was liked or retweeted), the total number of contributors (accounts creating tweets), and median original tweets per original contributor.

The content of all original, English-language tweets was analyzed independently by one of five authors (MP, SB, KW, NN, RP). To assess inter-rater reliability, a duplicate extraction of 100 tweets was performed by all extractors. We assigned each tweet to one of 13 pre-determined ‘content categories’ created after consensus discussion between authors: 1) peer-reviewed original research study related to Covid-19, excluding editorials, commentaries, or perspective articles; 2) editorial, commentary, or perspective article published in a journal or repository relevant to Covid-19, including scientific article pre-prints; 3) FOAM resources pertaining to the care of Covid-19 patients; 4) public health agency website or university website (e.g. Centers for Disease Control and Prevention); 5) medical podcast or video relevant to Covid-19; 6) personal or learned experience caring for Covid-19 patients; 7) a statement or discussion refuting blatantly false or misleading information regarding Covid-19; 8) a discussion about policy or public health measures related to Covid-19; 9) a discussion of the personal or emotional impact of Covid-19; 10) a tweet that provided blatantly false or misleading information (defined through consensus agreement by two senior authors (RP and RU) that the tweet contained false or misleading information based on current medical consensus); 11) a tweet asking for advice or for others to share experience caring for Covid-19 patients; 12) other Covid-19 related tweets that did not fit in the other categories; 13) non-Covid-19-related tweets. The final category was included for tweets that used the aforementioned hashtags but mentioned content entirely unrelated to Covid-19. The overall best fitting category was selected if multiple classifications were possible, and consensus discussion was allowed if needed. Once categorized, we calculated the number and percentage of tweets in each content category by day and week.

Contributors, original research, and FOAM content

We determined the demographics of Covid-19 FOAM contributors by reviewing 1000 random profiles of the contributors whose tweets were captured in the search strategy, extracting: the number of followers, total tweets, country of residence, and contributor source (institution, nurse, staff physician, resident physician, medical student, respiratory therapist, pharmacist, other healthcare professional, non-healthcare professional, non-clinician researcher, healthcare-related group). The profiles were randomized and selected using the randomize function in Microsoft Excel (Microsoft Corporation, Redmond, WA). We performed a similar analysis on the contributor profiles whose tweets were flagged as blatantly false or misleading.

To evaluate the dissemination of original research via Twitter, we analyzed the journal of publication, country of the corresponding author, article type (epidemiological study, intervention study, diagnostic study, basic science, case series, or other), and the median number of days between online publication (either pre-publication or online) and tweet for each included article. To ensure a focus on new research being conducted on Covid-19 (as opposed to previous coronavirus infections), we excluded research articles published before 2020.

We also identified tweets that linked to FOAM resources and the source (website), type of resource, number of tweets, including the resource, and the median number of days between FOAM publication online and the tweet.

Data analysis

We saved the extracted data in Microsoft Excel 2013 (Microsoft Corporation) and analyzed it using R version 3.6.2 (R Project for Statistical Computing, R Core Team, Vienna, Austria). When appropriate, we assessed the distribution of our data using a Shapiro-Wilks test and calculated the mean (+/- standard deviation) for normally distributed data and median (1st and 3rd interquartile range) for data that were not normally distributed. A post-hoc Mann-Whitney U test was performed to compare the days between publication of original research or FOAM resource and tweet. A post-hoc Mann-Kendall trend test with a Bonferroni correction was used to assess for a trend in the percentage of total tweets represented by each content category per week. Inter-rater reliability was assessed using Fleiss’ Kappa. Statistical significance was set at a p-value of less than or equal to 0·05.

## Results

**Figure 1:**
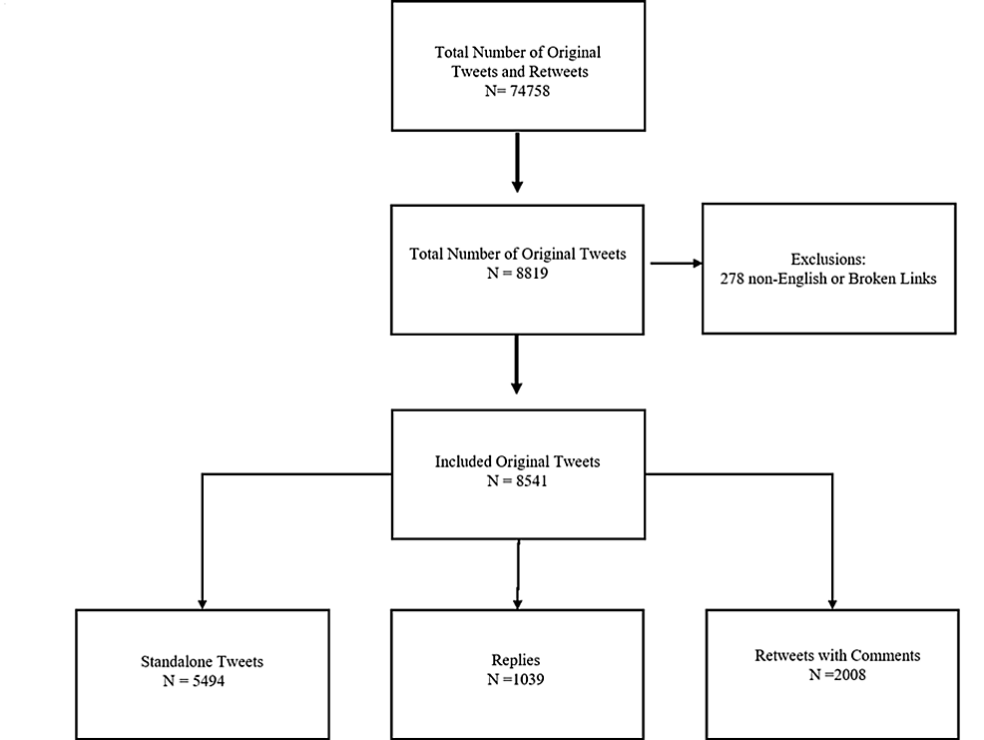
Flow diagram from included tweets

The first tweet matching the search criteria was on January 19, 2020, and from then until March 21, 2020, 74,758 original tweets and retweets from 52,917 contributors were created. Of these, 8819 (11.8%) were original tweets created by 4104 contributors, and 65,490 (88.2%) were retweets (Table 1). We excluded 278 tweets because they were not written in English or contained broken links. Of the remaining 8541 (11.4%) original tweets, 5494 were standalone tweets, 1039 were replies, and 2008 were retweets with comments (Figure 1). The original tweets and retweets reached 95,072,663 Twitter users and had a total of 388,701 likes or replies. Contributors to original tweets had a median number of 489 (interquartile range (IQR) 144, 1601) followers and published a median of one (IQR 1, 2) original tweet. A Shapiro-Wilks test showed the data was non-parametric (p < 0.001).

**Table 1:** Characteristics of total tweets, retweets, and contributors

Characteristics	Number (%)
Original tweets and retweets (total)	74,758 (100%)
Retweets without comments	65,940 (88·2%)
Original tweets (included)	8541 (11·4%)
-Standalone tweets	5494
-Replies	1039
-Retweets with comments	2008
Median number of original tweets per original contributor (IQR)	1 (1.2)
Total reach (number of unique people who saw the tweet)	95,072,663
Total impressions (number of likes and retweets)	388,701
Total contributors (tweets and retweets)	52,917
Original contributors	4104
Median followers per original contributor (IQR)	489 (IQR 144, 1601)
Language (tweets and retweets)	
English	72,927 (97.6%)
Unclassified	909 (1.2%)
Spanish	560 (0.7%)
German	124 (0.2%)
French	45 (0.1%)

Content analysis

**Figure 2:**
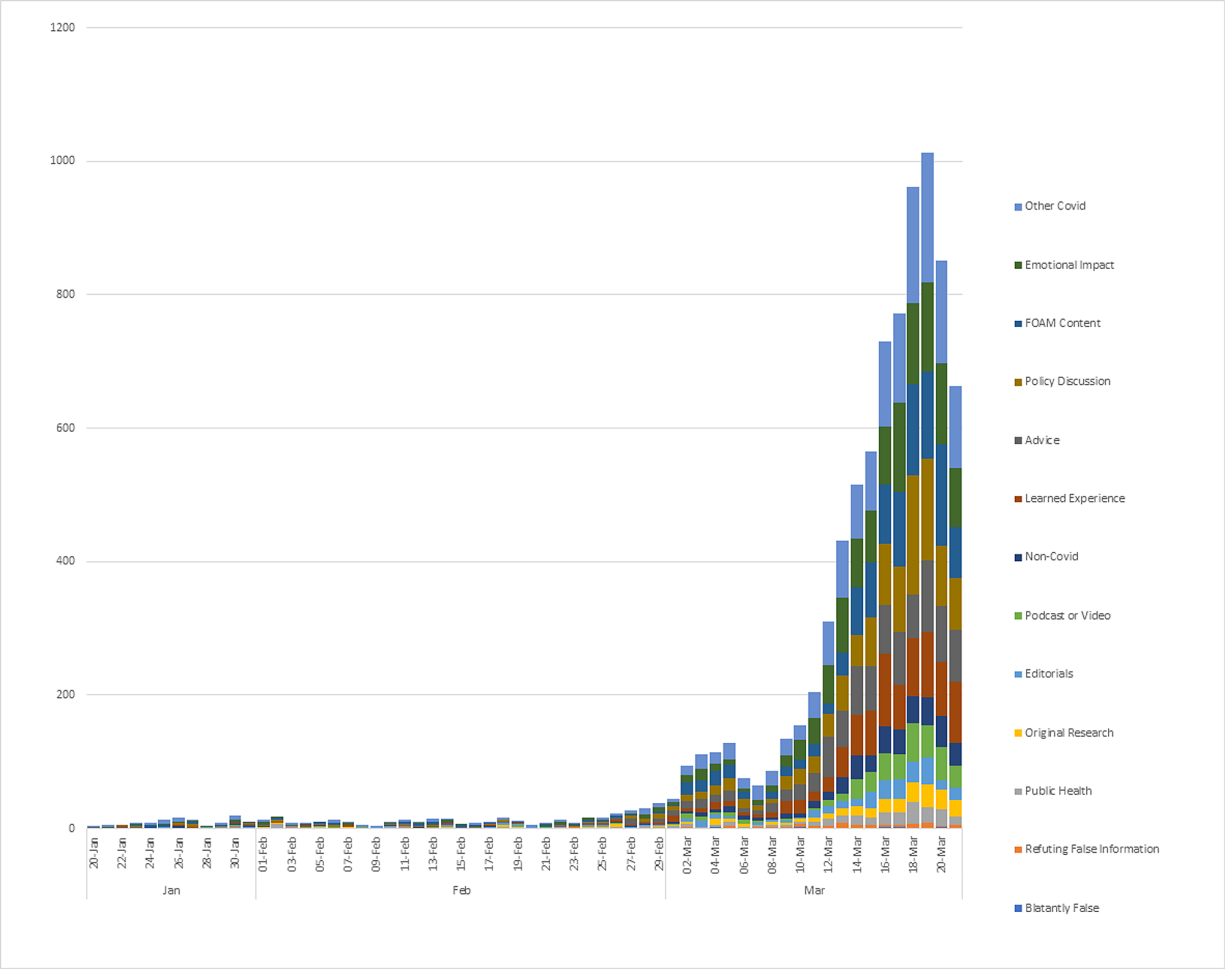
Total number of original tweets by content category per week

The number of tweets per day in each content category is displayed in Figure 2. The tweets per week are displayed in Appendix 1. Most Covid-19-related tweets (1557 or 18.2%) did not fall into one of the pre-determined categories. There were 1122 tweets sharing FOAM resources, including blog posts (13.1%), 275 sharing peer-reviewed original research articles (3.2%), 304 sharing non-research journal articles (editorials, commentaries, or perspectives) (3.6%), 410 sharing podcasts or videos (4.8%), and 245 linking to public health agencies or university websites (2.9%). There were also 1190 tweets discussing emotional impact (13.9%), 1111 tweets discussing public policy (13.0%), 928 tweets asking for advice pertaining to Covid-19 patients (10.9%), and 873 tweets about learned or personal experience caring for Covid-19 patients (10.2%). In addition, there were 83 tweets refuting false information (1.0%), 19 tweets providing blatantly false or misleading information (0.2%), and 424 non-Covid-19-related tweets (5.0%). There was no significant change in the percentage of total tweets represented by each content category over time (see supplementary materials). Overall, interrater reliability was fair with a kappa of 0.31. A selection of representative tweets is provided in Table 2.

**Table 2:** Sample of original tweets included in the study

Content Category	Tweet
Link to FOAM resources	Free Critical care training modules from @SCCM for non-ICU clinicians to prepare #medtwitter #COVID19 #covid4MDs
Asking for advice	Hi, primary care doctor at a major NYC hospital here. Now that’s there’s community transmission of COVID-19, how do we treat patients with mild respiratory symptoms(cough and fever) who have not had contact with known COVID-19 cases? #medtwitter #COVIDー19 @CDCgov 1/5
Link to original research	Remdesivir and chloroquine effectively inhibit the recently emerged novel coronavirus (2019-nCoV) in vitro https://t.co/WIBwkSNS9n #coronavirus #covid19 #medtwitter #internalmedicine
Blatantly false information	REPORT: #CoronaVirus is a bio-weapon experiment gone wrong, suspect global experts. #breakingnews #medtwitter #covid19 #wuhan #china
Other Covid-19 related discussion	1) Randomized clinical trials are important. Promising doesn’t mean it will work. We have seen other promising medicines fail in trials. 2) We have a chloroquine shortage. MGH has been using hydroxychloroquine instead. #medtwitter #covid19
Sharing personal experience	Reminder that how you take OFF PPE is very important so u don’t shower yourself w virus. Clutch gown at chest, pulls down over gloves, pull off with gloves, wash hands, pull off mask and goggles straight away from face (NOT UP); wash hands. #COVID19 #coviddoc #medtwitter
Emotional Impact	As the world is shaken, it turns to us. We can’t be shaken. We have an opportunity to be the beacon that guides the world. That’s rare and wonderful. In midst of uncertainty/fear, there is no other place I’d rather be. Proud to be in #healthcare #COVID19 #medtwitter #intensivist

Contributor demographics

The one-thousand randomly sampled contributors published 2464 tweets, which were cast to a total of 2,618,061 followers (Table 3). Their median number of followers was 455 (IQR 136, 1582). Contributors included 437 staff physicians (43.7%), 77 resident physicians (7.7%), 73 non-healthcare professionals (7.3%), 68 medical students (6.8%), and 78 nurses and other healthcare professionals (7.8%), and 126 (12.6%) indeterminate. Of the 1000 randomly-sampled contributors, 614 were from North America (61.4%), 100 were from Europe (10.0%), 26 were from Australia or New Zealand (2.6%), 24 were from Asia (2.4%), six were from South America (0.6%), five were from the Middle East (0.5%), four were from Africa (0.4%), and 221 (22.1%) indeterminate.

**Table 3:** Demographics of 1000 randomly selected contributors

Source/Profession	Number (%)
Physician	437 (43.7)
Not Reported	126 (12.6)
Institution or Organization	114 (11.4)
Resident Physician	77 (7.7)
Non-healthcare professional	73 (7.3)
Medical student	68 (6.8)
Other Healthcare Professional	45 (4.5)
Nurse	31 (3.1)
Non-clinician researcher	27 (2.7)
Pharmacist	2 (0.2)
Location of Contributor	
North America	614 (61.4)
Not Reported	221 (22.1)
Europe	100 (10.0)
Australia or New Zealand	26 (2.6)
Asia	24 (2.4)
South America	6 (0.6)
Middle East	5 (0.5)
Africa	4 (0.4)

Of the 19 contributors who produced tweets flagged as blatantly false or misleading, two (10.5%) were staff physicians, three (15.8%) were organization or institutions, two were nurses (10.5%), one (5.3%) was a non-clinician researcher, one (5.3%) was a non-healthcare professional, and 10 (47.4%) were undetermined. Their median number of followers was 728 (IQR 189, 1145).

Dissemination of original research

There were 275 tweets that linked to 157 unique peer-reviewed original research articles. Of these, 23 were published before January 1, 2020, and were excluded from the analysis. The 134 remaining studies included 18 epidemiological studies (13.4%), four intervention studies (3.0%), four diagnostic studies (3.0%), 10 basic science studies (7.5%), and 98 case series or other (73.1%). The top five most common countries of corresponding authors were China (57, 42.5%), the United States of America (29, 21.6%), Australia (11, 8.2%), the United Kingdom (15, 11.2%), and Italy (8, 6.0%). The top five most common journals of publication were The Lancet (20, 14.9%), The Journal of the American Medical Association (18, 13.4%), The Lancet Respiratory Medicine (9, 6.7%), The New England Journal of Medicine (8, 6.0%), and The Medical Journal of Australia (8, 6.0%). The median number of days between publication and tweet was 2 (IQR 1, 5). Appendix 2 lists the individual studies.

Dissemination of FOAM resources

There were 1122 tweets containing links to FOAM resources (websites, blogs, infographics, or attached files). The top 10 FOAM resources included in original tweets were “onepagericu.com” (47, 4.2%), “emcrit.org” (32, 2.9%), “esicm.org” (28, 2.5%), “sccm.org” (23, 2.0%), “butterflynetwork.com” (17, 1.5%), “rebelem.com” (13, 1.2%), “elso.blog” (12, 1.1%) “insightplus.mja.com.au” (12, 1.1%), “ultrasoundtraining.com.au" (12, 1.1%), “intensiveblog.com" (11, 1.0%), and “propofology.com” (11, 1.0%). The median number of days from publication of a FOAM resource to dissemination on Twitter was one (IQR 0, 3). Many of the resources that were shared were singular attachments that did not contain a link to a FOAM website, blog, or resource. These included 79 documents (eg, hospital Covid-19 protocols), 83 unsourced infographics, and 27 webinars (including participant notes). The median number of days between publication of FOAM resources and the tweet was shorter than for original peer-reviewed research (1 vs. 2, p < 0·0001).

## Discussion

Covid-19 is not the first pandemic where Twitter has played an important role in sharing information. The H1N1 pandemic and Ebola epidemic were widely discussed on Twitter, with millions of original tweets [12-13]. When compared to the H1N1 pandemic in 2009, however, the number of tweets discussing Covid-19 tweets has increased exponentially, surging in early March 2020 (Figure 2) [13]. For context, at that time, Europe had Covid-19 cases across the continent, with several hundred deaths in Italy alone [14]. North America also had its first significant outbreak in Washington, USA [15]. Although the FOAM community has contributors worldwide, most tweets were created by North American contributors, which may account for the temporal association between Twitter use and western outbreaks of Covid-19.

Given the immediacy and reach of social media, the FOAM community may be ideally situated to share medical resources during a pandemic. We found that more than one in four tweets contained a link to a Covid-19 resource. Compared to traditional publication peer review, the publication and editorial processes of FOAM resources vary [10,16]. FOAM relies on transparent and open post-publication peer review where other contributors can discuss, critique, and sometimes even contribute to resources [7]. One illustration of effective post-publication peer review during the Covid-19 pandemic has been the Internet Book of Critical Care (IBCC) chapter on Covid-19 [17]. From March 2 to April 16, 2020, the IBCC received over 2·1 million views, with over 180 comments contributing to post-publication peer review [18]. This has led to numerous revisions of the chapter to incorporate new evidence. To consolidate and share the vast amount of information being generated during a pandemic, open post-publication peer review may help balance the timely dissemination of content whilst ensuring its accuracy and quality.

In addition to the speed and reach of FOAM publication, social media may be particularly effective in sharing tacit knowledge [9]. This stems in part from its ability to facilitate discussions and story-telling, which are key components of tacit knowledge translation [9]. During Covid-19, the geographic progression of the disease from Asia, to Europe, to North America allowed for clinicians to share their experiences managing Covid-19 patients. In our study, we found that 9.8% percent of original tweets shared learned experiences and 10·8% represented individuals asking for crowd-sourced advice. Many of these early tweets pertained to the diagnosis of Covid-19 infection, ultrasound use, airway management, personal protective equipment, and mechanical ventilation. These resources may have helped clinicians and organizations to prepare for Covid-19.

With the unprecedented amount of new Covid-19 research being published, it seems increasingly difficult to find accurate and reliable content online. This has been termed an “infodemic’ by the WHO [19]. Although Twitter might contribute to this through the indiscriminate sharing of information, it can also flag important new research and give clinicians a forum to openly critique it. In our study, we found that contributors rapidly tweeted new Covid-19 research, with a median time between publication and tweet of two days. The majority were published in high-impact journals and included important early studies on Covid-19. The immediacy of discussion and rebuttal provided through Twitter also may be valuable, especially when many studies are first being published as pre-prints and have not undergone peer review. For example, when Gautret et al. published their initial pre-print advocating for the use of hydroxychloroquine and azithromycin as a treatment for Covid-19 [20], many FOAM contributors called for more rigorous studies with patient-important outcomes before widespread adoption (Table 2). Their criticisms were substantiated with recent studies showing increased adverse events and a potential association with mortality for hydroxychloroquine [21-22].

Contributing to and participating in the FOAM community is not without risks, and the unwritten rule is caveat emptor (buyer beware). Across social media, the potential for receiving misinformation is real and significant [23]. In this study, 19 contributors contributed blatantly false or misleading information; however, this represented only 0·2% of the total number of tweets in this analysis. Whereas blatantly misleading tweets are relatively easy to identify, a significant concern is when a reader is misled through either misrepresentation of opinion as fact, sensational anecdotes, or providing content without context. Furthermore, subtly misleading or incorrect information may prove much more challenging to interpret and is a serious and ongoing concern when using open-sourced information. A study by Kouzy et al. found that one in four tweets about Covid-19 across Twitter (no FOAM hashtags) contained misinformation [24]. We suspect the rate of subtle misinformation in the FOAM community is higher than the 0·2% found in this study; however, given that the community’s collective goal is to share legitimate knowledge, it is likely lower than the broader Twitter community.

FOAM has the potential to decrease the knowledge translation gap during Covid-19; however, resources may be of variable quality [25]. Readers are responsible for critically appraising online content; however, locating quality resources, to begin with, can be a challenge. The Social Media Index (SMI) provides a list of FOAM websites that are both impactful [26] and high quality [27], analogous in some ways to a journal’s impact factor. When reading these resources, the Academic Life in Emergency Medicine (ALiEM) AIR tool or revised Medical Education Translational Resources: Impact and Quality (METRIQ) tool has been validated to help determine the quality of content [28-29]. Readers may use these tools as an aid when navigating unfamiliar FOAM resources to better appraise the quality of the online resource.

Despite potential pitfalls from engaging with the FOAM community, it is a vibrant community that contributes to the timely dissemination and translation of medical knowledge throughout the world. Few other media, if any, are capable of sharing information, crowdsourcing advice, and providing warnings more rapidly; again, supported by the fact that new articles and FOAM resources discussing Covid-19 were shared within several days of online publication. In situations where efficient communication is essential, such as in global pandemic, natural disasters, or political upheavals, the benefits of such a system may outweigh the possibility of misleading information. It is also important to recognize that papers published in traditional print journals are not immune to misleading information and bias, and may have their own ‘Spin’ or biased interpretation of results. Furthermore, Twitter allows for tailored advice to individuals through the ability to share personal experiences and ask specific questions, as was seen in our study. With these potential benefits, we feel that Twitter FOAM augments knowledge translation achieved through conventional scientific publications and will continue to do so in the years to come.

Limitations

Our study has several limitations. Although a broad hashtag search strategy was used, some FOAM hashtags were not included. As well, many Twitter contributors do not add hashtags to all of their Tweets, meaning that some tweets that would have been relevant were missed. For example, it is possible that certain tweets with relevant medical information containing only Covid-19-related hashtags could have been missed. Additionally, although the search was up to date as of March 21, 2020, the necessary time for analysis and manuscript preparation means it does not reflect current Twitter use. As well, inter-rater reliability was only fair, likely related to the fact some tweets fell into several categories. This may account for the reason why a fair proportion of tweets were classified as “other Covid-19.” Finally, during the initial months of Covid-19, the largest number of cases were in Asia and Europe [1,30], yet the study was limited to English tweets, potentially selecting for a North American or Anglo-biased perspective.

## Conclusions

In the age of social media, many clinicians use Twitter to share resources and ideas with the goal of improving care for their patients. Twitter is effective in disseminating information; however, it comes with challenges in ensuring content is accurate and relevant. This study provides a detailed characterization of early medical tweets during the Covid-19 pandemic and represents a first step in understanding Twitter use among the FOAM community during the Covid-19 pandemic. Further work is required to improve Twitter as a knowledge translation tool both for Covid-19 and future global crises, such that misinformation and bias is minimized and factual knowledge dissemination maximized. Covid-19 has united clinicians around the world, and perhaps more than ever, effective strategies for sharing new ideas, accurate information, and quality research are needed.

## Appendices

Appendix 1 

**Table 4:** Number of original tweets by content category per week

	Week 1	Week 2	Week 3	Week 4	Week 5	Week 6	Week 7	Week 8	Week 9	Total Tweets	P value*
Original Research	1 (1.6%)	2 (2.3%)	5 (8.1%)	2 (2.6%)	7 (9.1%)	10 (5.1%)	28 (4.1%)	61 (2.6%)	159 (3.2%)	275	0.47
Editorials	2 (3.3%)	6 (6.9%)	6 (9.7%)	7 (9.1%)	8 (10.4%)	12 (6.1%)	27 (4.0%)	74 (3.2%)	162 (3.2%)	304	0.25
FOAM Content	7 (11.5%)	14 (16.1%)	9 (14.5%)	17 (22.1%)	10 (13.0%)	14 (7.1%)	104 (15.4%)	252 (10.9%)	695 (13.9%)	1122	0.6
Public Health	1 (1.6%)	8 (9.2%)	1 (1.6%)	3 (3.9%)	3 (3.9%)	13 (6.6%)	26 (3.9%)	57 (2.5%)	133 (2.7%)	245	0.83
Podcast or Video	2 (3.3%)	3 (3.4%)	4 (6.5%)	4 (5.2%)	5 (6.5%)	3 (1.5%)	35 (5.2%)	88 (3.8%)	266 (5.3%)	410	0.6
Learned Experience	4 (6.6%)	8 (9.2%)	5 (8.1%)	4 (5.2%)	4 (5.2%)	20 (10.2%)	50 (7.4%)	247 (10.7%)	531 (10.6%)	873	0.21
Refuting False Informatino	0 (0.0%)	4 (4.6%)	4 (6.5%)	2 (2.6%)	2 (2.6%)	1 (0.5%)	10 (1.5%)	34 (1.5%)	26 (0.5%)	83	0.29
Policy Discussion	7 (11.5%)	13 (14.9%)	5 (8.1%)	10 (13.0%)	6 (7.8%)	26 (13.3%)	79 (11.7%)	275 (11.9%)	690 (13.8%)	1111	0.6
Emotional Impact	7 (11.5%)	9 (10.3%)	7 (11.3%)	8 (10.4%)	9 (11.7%)	23 (11.7%)	67 (9.9%)	376 (16.2%)	684 (13.7%)	1190	0.18
Blatantly False	0 (0.0%)	0 (0.0%)	0 (0.0%)	0 (0.0%)	2 (2.6%)	1 (0.5%)	4 (0.6%)	4 (0.2%)	8 (0.2%)	19	0.23
Advice	6 (9.8%)	2 (2.3%)	1 (1.6%)	2 (2.6%)	3 (3.9%)	31 (15.8%)	74 (11.0%)	321 (13.9%)	488 (9.8%)	928	0.23
Other Covid	20 (32.8%)	13 (14.9%)	14 (22.6%)	14 (18.2%)	16 (20.8%)	32 (16.3%)	136 (20.1%)	404 (17.5%)	908 (18.2%)	1557	0.18
Non-Covid	4 (6.6%)	5 (5.7%)	1 (1.6%)	4 (5.2%)	2 (2.6%)	10 (5.1%)	35 (5.2%)	122 (5.3%)	241 (4.8%)	424	0.47
Total Tweets Per Week	61	87	62	77	77	196	675	2315	4991	8541	
*Man Kendall Test for Monotonic Trends											

Appendix 2 

**Table 5:** Research articles with Pubmed ID linked in original tweets

PMID	Study Title	Journal of Publication	Article Type*
25599463	Fluid Management With a Simplified Conservative Protocol for the Acute Respiratory Distress Syndrome	Critical Care Medicine	1
25903751	A cluster randomised trial of cloth masks compared with medical masks in healthcare workers	BMJ Open	1
29071828	Intactness of Medical Nonsterile Gloves on Use of Alcohol Disinfectants	Ann Lab Med	1
30336170	Comparison of high-flow nasal cannula versus oxygen face mask for environmental bacterial contamination in critically ill pneumonia patients: a randomized controlled crossover trial	Journal of Hospital Infection	1
31479137	N95 Respirators vs Medical Masks for Preventing Influenza Among Health Care Personnel	JAMA	1
32178954	Anesthetic Management of Patients with COVID 19 Infections during Emergency Procedures	Journal of Cardiothoracic & Vascular Anesthesia	1
32187464	A Trial of Lopinavir–Ritonavir in Adults Hospitalized with Severe Covid-19	NEJM	1
32205204	Hydroxychloroquine and azithromycin as a treatment of COVID-19: results of an open-label non-randomized clinical trial	International Journal of Antimicrobial Agents	1
32049601	Chest CT for Typical 2019-nCoV Pneumonia: Relationship to Negative RT-PCR Testing	Radiology	2
32166346	Findings of lung ultrasonography of novel coronavirus pneumonia during the 2019–2020 epidemic	Intensive Care Medicine	2
16115318	Chloroquine is a potent inhibitor of SARS coronavirus infection and spread	Virology Journal	3
17302372	Indomethacin has a potent antiviral activity against SARS coronavirus.	Antiviral therapy	3
17522231	Clathrin-Dependent Entry of Severe Acute Respiratory Syndrome Coronavirus into Target Cells Expressing ACE2 with the Cytoplasmic Tail Deleted	Journal of Virology	3
20430477	Inactivation of influenza A virus H1N1 by disinfection process.	Am J Infect Control	3
22436202	Cough aerosol in healthy participants: fundamental knowledge to optimize droplet-spread infectious respiratory disease management	BMC Pulmonary Medicine	3
25224111	Aerosol Dispersion During Various Respiratory Therapies: A Risk Assessment Model of Nosocomial Infection to Health Care Workers	Hong Kong Med J	3
28193191	Effective inhibition of MERS-CoV infection by resveratrol	BMC Infectious Diseases	3
29511076	Coronavirus Susceptibility to the Antiviral Remdesivir (GS-5734) Is Mediated by the Viral Polymerase and the Proofreading Exoribonuclease	American Society for Microbiology	3
29678452	Ultraviolet Germicidal Irradiation of Influenza-Contaminated N95 Filtering Facepiece Respirators	Am J Infect Control	3
31753725	Pro-inflammatory monocyte profile in patients with major depressive disorder and suicide behaviour and how ketamine induces anti-inflammatory M2 macrophages by NMDAR and mTOR	EBio Medicine	3
32020029	Remdesivir and chloroquine effectively inhibit the recently emerged novel coronavirus (2019-nCoV) in vitro	Cell Research	3
32075877	Cryo-EM structure of the 2019-nCoV spike in the prefusion conformation	Science	3
32150618	In Vitro Antiviral Activity and Projection of Optimized Dosing Design of Hydroxychloroquine for the Treatment of Severe Acute Respiratory Syndrome Coronavirus 2 (SARS-CoV-2)	Clinical Infectious Diseases	3
32182409	Aerosol and surface stability of HCoV-19 (SARS-CoV-2) compared to SARS-CoV-1	NEJM	3
32192578	COVID-19: consider cytokine storm syndromes and immunosuppression	The Lancet	3
32194981	Hydroxychloroquine, a less toxic derivative of chloroquine, is effective in inhibiting SARS-CoV-2 infection in vitro	Cell Discovery	3
32199469	Prolonged presence of SARS-CoV-2 viral RNA in faecal samples	Lancet Gastroenterology & Hepatology	3
32237278	Isolation and rapid sharing of the 2019 novel coronavirus (SARS-CoV-2) from the first patient diagnosed with COVID-19 in Australia	Medical Journal of Australia	3
25637115	Face touching: a frequent habit that has implications for hand hygiene.	Am J Infect Control	4
31585142	Mobile phones as fomites for potential pathogens in hospitals: microbiome analysis reveals hidden contaminants	Journal of Hospital Infection	4
31607599	Influenza vaccination and respiratory virus interference among Department of Defense personnel during the 2017-2018 influenza season.	Vaccine	4
32064853	The epidemiological characteristics of an outbreak of 2019 novel coronavirus diseases (COVID-19) in China	CMAPH	4
32105632	Clinical course and outcomes of critically ill patients with SARS-CoV-2 pneumonia in Wuhan, China: a single-centered, retrospective, observational study	Lancet Respiratory Medicine	4
32119825	Feasibility of controlling COVID-19 outbreaks by isolation of cases and contacts	Lancet Global Health	4
32167524	Risk Factors Associated With Acute Respiratory Distress Syndrome and Death in Patients With Coronavirus Disease 2019 Pneumonia in Wuhan, China	JAMA Internal Medicine	4
32171076	Clinical course and risk factors for mortality of adult inpatients with COVID-19 in Wuhan, China: a retrospective cohort study	Lancet	4
32171390	Real estimates of mortality following COVID-19 infection	Lancet	4
32179660	Epidemiology of COVID-19 Among Children in China	Pediatrics	4
32179701	Substantial undocumented infection facilitates the rapid dissemination of novel coronavirus (SARS-CoV2)	Science	4
32179890	Risk Factors of Healthcare Workers with Corona Virus Disease 2019: A Retrospective Cohort Study in a Designated Hospital of Wuhan in China	Clinical Infectious Diseases	4
15963157	The Impact of Severe Acute Respiratory Syndrome on Medical House Staff	J Gen Intern Med	5
16400088	Concept of Operations for Triage of Mechanical Ventilation in an Epidemic	Academic Emergency Medicine	5
16885402	A Single Ventilator for Multiple SimulatedPatients to Meet Disaster Surge	Academic Emergency Medicine	5
19773323	Physical 1s to interrupt or reduce the spread of respiratory viruses: systematic review	BMJ	5
23538558	Search for inhibitors of endocytosis	Cellular Logistics	5
28556555	Examination of Hydroxychloroquine Use and Hemolytic Anemia in G6PDH-Deficient Patients	Arthritis Care Res	5
28586113	Lung Consolidation Locations for Optimal Lung Ultrasound Scanning in Diagnosing Pediatric Pneumonia	J Ultrasound Med	5
30368986	Lung‐protective Ventilation for Acute Respiratory Distress Syndrome	Academic Emergency Medicine	5
31766051	Standard and Precordial Leads Obtained With an Apple Watch	Annals of Internal Medicine	5
31871560	Involving Physicians-in-Training in the Care of Patients During Epidemics	Journal of Graduate Medical Education	5
31967327	Emerging coronaviruses: Genome structure, replication, and pathogenesis	Journal of Medical Virology	5
31986264	Clinical features of patients infected with 2019 novel coronavirus in Wuhan, China	Lancet	5
32004427	First Case of 2019 Novel Coronavirus in the United States	NEJM	5
32022836	2019 Novel Coronavirus—Important Information for Clinicians	JAMA	5
32031570	Clinical Characteristics of 138 Hospitalized Patients With 2019 Novel Coronavirus–Infected Pneumonia in Wuhan, China	JAMA	5
32034323	Mechanisms of action of hydroxychloroquine and chloroquine: implications for rheumatology	Nature Reviews	5
32035030	The mental health of medical workers in Wuhan, China dealing with the 2019 novel coronavirus	Lancet	5
32052373	Practical recommendations for critical care and anesthesiology teams caring for novel coronavirus (2019-nCoV) patients	Canadian Journal of Anesthesia	5
32061335	Therapeutic and triage strategies for 2019 novel coronavirus disease in fever clinics	Lancet Respiratory Medicine	5
32066488	Validation of neuromuscular blocking agent use in acute respiratory distress syndrome: a meta-analysis of randomized trials	Critical Care	5
32066541	Cancer patients in SARS-CoV-2 infection: a nationwide analysis in China	Lancet Oncology	5
32074258	Preparing for the Most Critically Ill Patients With COVID-19 The Potential Role of Extracorporeal Membrane Oxygenation	JAMA	5
32077115	Clinical characteristics of 140 patients infected with SARS‐CoV‐2 in Wuhan, China	Allergy	5
32085846	Pathological findings of COVID-19 associated with acute respiratory distress syndrome	Lancet Respiratory Medicine	5
32085849	The first Vietnamese case of COVID-19 acquired from China	Lancet Infectious Diseases	5
32087116	Asymptomatic cases in a family cluster with SARS-CoV-2 infection	Lancet Infectious Diseases	5
32091533	Characteristics of and Important Lessons From the Coronavirus Disease 2019 (COVID-19) Outbreak in China	JAMA	5
32100024	De-isolating Coronavirus Disease 2019 Suspected Cases: A Continuing Challenge	Clinical Infectious Diseases	5
32101510	Correlation of Chest CT and RT-PCR Testing in Coronavirus Disease 2019 (COVID-19) in China: A Report of 1014 Cases	Radiology	5
32105633	Staff safety during emergency airway management for COVID-19 in Hong Kong	Lancet Respiratory Medicine	5
32109013	Clinical Characteristics of Coronavirus Disease 2019 in China	NEJM	5
32112714	The psychological impact of quarantine and how to reduce it: rapid review of the evidence	Lancet	5
32115364	Epidemiological and clinical characteristics of heart transplant recipients during the 2019 coronavirus outbreak in Wuhan china; a descriptive survey report	Journal of Heart and Lung Transplantation	5
32125362	Epidemiologic Features and Clinical Course of Patients Infected With SARS-CoV-2 in Singapore.	JAMA	5
32125452	Clinical predictors of mortality due to COVID-19 based on an analysis of data of 150 patients from Wuhan, China	Intensive Care Medicine	5
32125455	Angiotensin-converting enzyme 2 (ACE2) as a SARS-CoV-2 receptor: molecular mechanisms and potential therapeutic target	Intensive Care Medicine	5
32129805	Air, Surface Environmental, and Personal Protective Equipment Contamination by Severe Acute Respiratory Syndrome Coronavirus 2 (SARS-CoV-2) From a Symptomatic Patient	JAMA	5
32133578	Review of the Clinical Characteristics of Coronavirus Disease 2019 (COVID-19)	J Gen Intern Med	5
32134381	Lack of Vertical Transmission of Severe Acute Respiratory Syndrome Coronavirus 2, China	Emerging Infectious Diseases	5
32144127	Coronavirus disease 2019 (covid-19): a guide for UK GPs	BMJ	5
32145829	Respiratory support for patients with COVID-19 infection	Lancet	5
32146721	An outbreak of COVID‐19 caused by a new coronavirus: what we know so far	Medical Journal of Australia	5
32150360	Features, Evaluation and Treatment Coronavirus (COVID-19)		5
32150748	The Incubation Period of Coronavirus Disease 2019 (COVID-19) From Publicly Reported Confirmed Cases: Estimation and Application	Annals of Internal Medicine	5
32151335	Clinical characteristics and intrauterine vertical transmission potential of COVID-19 infection in nine pregnant women: a retrospective review of medical records	Lancet	5
32159735	Care for Critically Ill Patients With COVID-19	JAMA	5
32160316	Co‐infection of SARS‐CoV‐2 and HIV in a patient in Wuhancity, China	Journal of Medical Virology	5
32163697	Detection of Covid-19 in Children in Early January 2020 in Wuhan, China	NEJM	5
32166318	Protecting Health Care Workers During the COVID-19 Coronavirus Outbreak -Lessons From Taiwan's SARS Response	Clinical Infectious Diseases	5
32166346	Findings of lung ultrasonography of novel corona virus pneumonia during the 2019–2020 epidemic	Intensive Care Medicine	5
32167525	From Containment to Mitigation of COVID-19 in the US	JAMA	5
32167538	Critical Care Utilization for the COVID-19 Outbreak in Lombardy, Italy: Early Experience and Forecast During an Emergency Response	JAMA	5
32167853	Can Lung US Help Critical Care Clinicians in the Early Diagnosis of Novel Coronavirus (COVID-19) Pneumonia?	Radiology	5
32171062	Are patients with hypertension and diabetes mellitus at increased risk for COVID-19 infection?	Lancet Respiratory Medicine	5
32172175	Clinical considerations for patients with diabetes in times of COVID-19 epidemic	Diabetes & Metabolic Syndrome: Clinical Research & Reviews	5
32173110	A systematic review on the efficacy and safety of chloroquine for the treatment of COVID-19	Journal of Critical Care	5
32176257	COVID-19 and the Risk to Health Care Workers: A Case Report	Annals of Internal Medicine	5
32180175	Safety and efficacy of different anesthetic regimens for parturients with COVID-19 undergoing Cesarean delivery: a case series of 17 patients	Canadian Journal of Anesthesia	5
32180426	An Analysis of 38 Pregnant Women with COVID-19, Their Newborn Infants, and Maternal3 Fetal Transmission of SARS-CoV-2: Maternal Coronavirus Infections and Pregnancy Outcomes	Archives of Pathology & Laboratory Medicine	5
32181795	Coronavirus Disease 2019 (COVID-19) in Italy	JAMA	5
32182347	Perinatal Transmission of COVID-19 Associated SARS-CoV-2: Should We Worry?	Clinical Infectious Diseases	5
32187458	SARS-CoV-2 Infection in Children	NEJM	5
32191259	Characteristics and Outcomes of 21 Critically Ill Patients With COVID-19 in Washington State	JAMA	5
32192578	COVID-19: consider cytokine storm syndromes and immunosuppression	Lancet	5
32199075	Health security capacities in the context of COVID-19 outbreak: an analysis of International Health Regulations annual report data from 182 countries	Lancet	5
32199938	Catheterization Laboratory Considerations During the Coronavirus (COVID-19) Pandemic: From ACC’s 1al Council and SCAI	Journal of the American College of Cardiology	5
32203711	Planning and provision of ECMO services for severe ARDS during the COVID-19 pandemic and other outbreaks of emerging infectious diseases	Lancet Respiratory Medicine	5
32204907	The Novel Coronavirus 2019 epidemic and kidneys	Kidney International	5
32224769	Surviving Sepsis Campaign: Guidelines on the Management of Critically Ill Adults with Coronavirus Disease 2019 (COVID-19)	Intensive Care Medicine	5
32237278	Isolation and rapid sharing of the 2019 novel coronavirus (SARS-CoV-2) from the first patient diagnosed with COVID-19 in Australia	Medical Journal of Australia	5
32266965	COVID-19 precautions – easier said than done when patients are homeless	Medical Journal of Australia	5
32275288	Neurological Manifestations of Hospitalized Patients with COVID-19 in Wuhan, China: a retrospective case series study	N/A (medRxiv)	5
*Category: 1 = Intervention 2 = diagnostic 3 = basic science 4 = epidemiological 5= case studies, series, or other			

## References

[1] Grasselli G, Zangrillo A, Zanella A (2020). Baseline characteristics and outcomes of 1591 patients infected with SARS-CoV-2 admitted to ICUs of the Lombardy Region, Italy. JAMA.

[2] Zhou F, Yu T, Du R (2020). Clinical course and risk factors for mortality of adult inpatients with COVID-19 in Wuhan, China: a retrospective cohort study. Lancet.

[3] Bedford J, Enria D, Giesecke J (2020). COVID- 19: towards controlling of a pandemic. Lancet.

[4] Peyrin-Biroulet L (2020). Will the quality of research remain the same during the COVID-19 pandemic?. Clin Gastroenterol Hepatol.

[5] Rosenberg H, Syed S, Rezaie S (2020). The Twitter pandemic: the critical role of Twitter in the dissemination of medical information and misinformation during the COVID-19 Pandemic. CJEM.

[6] Thoma B, Chan TM, Paterson QS, Milne WK, Sanders JL, Lin M (2015). Emergency medicine and critical care blogs and podcasts: establishing an international consensus on quality. Ann Emerg Med.

[7] Cadogan M, Thoma B, Chan TM, Lin M (2014). Free Open Access Meducation (FOAM): the rise of emergency medicine and critical care blogs and podcasts (2002-2013). Emerg Med J.

[8] Thoma B, Joshi N, Trueger NS, Chan TM, Lin M (2014). Five strategies to effectively use online resources in emergency medicine. Ann Emerg Med.

[9] Panahi S, Wastson J, Partridge H (2020). Potentials of social media for tacit knowledge sharing among clinicians: preliminary findings. Dec 3.

[10] Azim A, Beck-Esmay J, Chan TM (2018). Editorial processes in Free Open Access Medical Educational (FOAM) resources. AEM Educ Train.

[11] von Elm E, Altman DG, Egger M (2007). Strengthening the Reporting of Observational Studies in Epidemiology (STROBE) statement: guidelines for reporting observational studies. BMJ.

[12] Odlum M, Yoon S (2015). What can we learn about the Ebola outbreak from tweets?. Am J Infect Control.

[13] Chew C, Eysenbach G (2010). Pandemics in the age of Twitter: content analysis of Tweets during the 2009 H1N1 outbreak. PLoS One.

[14] World Health Organization (2020). Coronavirus disease 2019 (COVID-19). Situation report - 48. World Health Organization. 2019 (COVID- 19): Situation Report 48. Geneva, World Health Organization, 2020.

[15] Arentz M, Yim E, Klaff L, Lokhandwala S, Riedo FX, Chong M, Lee M (2020). Characteristics and outcomes of 21 critically ill patients with COVID-19 in Washington State. JAMA.

[16] Sidalak D, Purdy E, Luckett-Gatopoulos S, Murray H, Thoma B, Chan TM (2017). Coached peer review: developing the next generation of authors. Acad Med.

[17] (2020). Internet book of critical care. https://emcrit.org/ibcc/covid19/.

[18] Farkas J (2020). Farkas, J., IBCC Covid-19 website traffic. Personal correspondance. https://emcrit.org/pulmcrit/covid19/.

[19] World Health Organization (2020). WHO. Novel coronavirus(2019-nCoV). Situation report - 13. 2019 (COVID- 19): Situation Report 13. Geneva, World Health Organization, 2020.

[20] Gautret P, Lagier JC, Parola P (2020). Hydroxychloroquine and azithromycin as a treatment of COVID- 19: results of an open-label non-randomized clinical trial. Int J Antimicrob Agents.

[21] Chen Z, Hu J, Zhang Z (2020). Efficacy of hydroxychloroquine in patients with COVID- 19: results of a randomized clinical trial [PREPRINT]. MedRxIV.

[22] Magagnoli JN, Siddharth N, Pereira F, Cummings T, Hardin JW, Sutton S, Ambati J (2020). Outcomes of hydroxychloroquine usage in United States veterans hospitalized with Covid-19. Med.

[23] Dijkstra S, Kok G, Ledford JG, Sandalova E, Stevelink R (2018). Possibilities and pitfalls of social media for translational medicine. Front Med.

[24] Kouzy R, Abi Jaoude J, Kraitem A (2020). Coronavirus goes viral: quantifying the COVID-19 misinformation epidemic on Twitter. Cureus.

[25] Grock A, Bhalerao A, Chan TM, Thoma B, Wescott AB, Trueger NS (2019). Systematic Online Academic Resource (SOAR) review: renal and genitourinary. AEM Educ Train.

[26] Thoma B, Sanders JL, Lin M, Paterson QS, Steeg J, Chan TM (2015). The social media index: measuring the impact of emergency medicine and critical care websites. West J Emerg Med.

[27] Thoma B, Chan TM, Kapur P (2018). The Social Media Index as an indicator of quality for emergency medicine blogs: a METRIQ study. Ann Emerg Med.

[28] Chan TM, Grock A, Paddock M, Kulasegaram K, Yarris LM, Lin M (2016). Examining reliability and validity of an online score (ALiEM AIR) for rating Free Open Access Medical Education resources. Ann Emerg Med.

[29] Colmers-Gray IN, Krishnan K, Chan TM (2019). The revised METRIQ score: a quality evaluation tool for online educational resources. AEM Educ Train.

[30] Guan WJ, Ni ZY, Hu Y (2020). Clinical characteristics of coronavirus disease 2019 in China. N Engl J Med.

